# Clinical Versus Pathological Staging in Patients with Resected Ground Glass Pulmonary Lesions

**DOI:** 10.3390/diagnostics14242874

**Published:** 2024-12-20

**Authors:** Dan Levy Faber, Abed Agbarya, Andrew Lee, Yael Tsenter, Sonia Schneer, Yulia Robitsky Gelis, Ronen Galili

**Affiliations:** 1 Department of Cardiothoracic Surgery, Lady Davis Carmel Medical Center, 7 Michal St., Haifa 3436212, Israel; sonias3@clalit.org.il (S.S.); ronenga@clalit.org.il (R.G.); 2 Rappaport Faculty of Medicine, Technion-Israel Institute of Technology, Haifa 3109601, Israel; 3 Oncology Institute, Bnai-Zion Medical Center, Haifa 3339419, Israel; 4 Department of Anesthesia, Lady Davis Carmel Medical Center, 7 Michal St., Haifa 3436212, Israel; andrewlee241088@gmail.com; 5 Pathology Institute, Lady Davis Carmel Medical Center, Haifa 3436212, Israel; yaeltse@clalit.org; 6 Pulmonary Division, Lady Davis Carmel Medical Center, Haifa 3436212, Israel; 7 Oncology Institute, Lin Medical Center and Carmel Medical Center, Haifa 3515210, Israel; yuliaro1@clalit.org.il

**Keywords:** radiological clinical staging, ground glass nodule, lung lesions, pathological staging, tumor classification, non-small cell lung cancer

## Abstract

Background: A ground glass nodule (GGN) is a radiologically descriptive term for a lung parenchymal area with increased attenuation and preserved bronchial and vascular structures. GGNs are further divided into pure versus subsolid lesions. The differential diagnosis for GGNs is wide and contains a malignant possibility for a lung adenocarcinoma precursor or tumor. Clinical and pathological staging of GGNs is based on the lesions’ solid component and falls into a specific classification including T0 for TIS, T1mi for minimally invasive adenocarcinoma (MIA) and T1abc for lepidic predominant adenocarcinoma (LPA) according to the eighth edition of the TNM classification of lung cancer. Correlation between solid parts seen on a CT scan and the tumor pathological invasive component is not absolute. Methods: This retrospective study collected the data of 68 GGNs that were operated upon in Carmel Medical Center. A comparison between preoperative clinical staging and post-surgery pathological staging was conducted. Results: Over a third of the lesions, twenty-four (35.3%), were upstaged while only four (5.9%) lesions were downstaged. Another third of the lesions, twenty-three (33.8%), kept their stage. In three (4.4%) cases, premalignant lesion atypical adenomatous hyperplasia (AAH) was diagnosed. Ten (14.7%) cases were diagnosed as non-malignant on final pathology. These findings show an overall low agreement between the clinical and pathological stages of GGNs. Conclusions: The relatively high percentage of upstaging tumors detected in this study and the overall safe and short surgical procedure advocate for surgical resection even in the presence of a significant number of non-malignant lesions that retrospectively do not mandate intervention at all.

## 1. Introduction

Lung cancer today is still the leading cause of cancer-related mortality worldwide with over 1.8 million deaths and close to 2.5 million new cases in 2022 [1]. One of the main causes for this high mortality rate is that most cases are diagnosed at an advanced stage. According to American National Cancer Institute statistics, only 21.6% of lung cancer patients are diagnosed at a localized (early) disease stage [2]. Publication of the groundbreaking National Lung Screening Trial (NLST) study, which for the first time demonstrated a reduction of 20.3% in the relative risk of mortality with Low-Dose Computed Tomography (LDCT) screening [3], started a new era of lung cancer screening. The cornerstone of lung cancer detection and screening is the CT modality. Usually, the malignant lung lesion appears on the scan as a spiculated solid nodule in the lung parenchyma with or without lymph node involvement [4]. However, other lesions may also be identified and raise suspicion. One common finding is a ground glass opacity (GGO) appearance nodule. A ground glass nodule (GGN) is a radiologically descriptive term for a lung parenchymal area with increased attenuation and preserved bronchial and vascular structures [5]. These GGNs are further divided into pure versus subsolid lesions. Nodules that are not visible on thin sections with a soft tissue, mediastinal CT window setting as opposed to a lung window setting are regarded as pure GGNs, while nodules that are partially visible on a mediastinal window setting are considered as subsolid lesions [6]. The differential diagnosis for GGN is wide (lung edema, interstitial lung disease, lung fibrosis, alveolar changes, inflammations and more), and, unfortunately, it contains a malignant possibility for a lung adenocarcinoma precursor [7,8,9,10]. The incidence of lung cancer presented as a GGN can reach as high as 63% [11,12]. On pathologic evaluation, most of those lesions have a lepidic pattern of growth, characterized by tumor cell proliferation along the alveoli walls. This growth pattern usually correlates with the level of haziness seen on a CT scan and the size of the solid component of the lesion; hence, the larger the solid component, the higher the invasiveness of the lesion [13,14]. The relation between the components of a GGN can be presented as the ratio between the solid component size and the overall tumor size known as the consolidation to tumor ratio (CTR).

In 2015, WHO classification adopted new entities of adenocarcinoma in situ (AIS), minimally invasive adenocarcinoma (MIA) and lepidic predominant adenocarcinoma (LPA). The definition of those early adenocarcinomas is based upon an entire histologic sampling of the tumor. Adenocarcinoma in situ (AIS) is an adenocarcinoma tumor of less than 3 cm of pure lepidic growth without any kind of invasive component or spread through air spaces. Nuclear atypia is absent or inconspicuous. Minimally invasive adenocarcinoma (MIA) is an adenocarcinoma tumor of less than 3 cm with predominantly lepidic growth, which has an invasive component of less than 5 mm in any one focus. The invasive component consists of either area of histological subtypes other than lepidic or myofibroblastic stromal tumor cell infiltration. Furthermore, the presence of lymphatics, blood vessels or pleura invasion, spread through air spaces (STAS) or tumor necrosis, preclude this definition. If the tumor is less than 3 cm with lepidic growth and has an invasive component of more than 5 mm, then the terms lepidic predominant adenocarcinoma (LPA) or adenocarcinoma with lepidic component are used. Tumor invasion into lymphatics, blood vessels or pleura and the presence of tumor necrosis also support this diagnosis [15,16,17,18].

This new concept of very early and, in some cases, noninvasive adenocarcinomas raised a difficulty in how to measure and stage those lesions [16]. Travis et al. in their proposals for coding T categories for subsolid nodules for the eighth edition of the TNM classification of lung cancer addressed this issue and set the foundations for GGN measuring and staging. The eighth edition TNM staging system manual states that for part solid adenocarcinomas, tumor size measurement, which is the T descriptor for clinical staging, is the solid component on a CT scan, and for pathological staging, it is the tumor invasive component on histology since those sizes determine the patient’s prognosis [19]. It is also recommended to document the full tumor size (ground glass plus solid components on a CT scan and lepidic growth plus invasive components on histology). Clinical and pathological staging of GGNs fall into a specific classification including T0 for TIS, T1mi for MIA and T1abc for LPA [19].

Still, researchers emphasize that the correlation between the solid component seen on a CT scan and the tumor pathological invasive component is not absolute and, in some cases, could represent a benign process such as a scar [16,20]. This emphasizes the importance of tumor stage revision between clinical and pathological staging. The current study aims to investigate this aspect of GGN evaluation. GGNs as early-stage lung cancer tumors are a relatively new field in the lung cancer staging system. Only in the last eighth edition of the lung cancer staging system were these tumors given the definition of T0 or T1 tumors. The clinical evaluation follow-up and approach of this lesion is complex due to the wide differential diagnosis and slow progression of this tumor. Clinical to pathological correlation may be less accurate considering the lesion’s obscure nature on imaging [21,22,23]. Evaluating this weakness, as performed in the current study, is important for clinicians’ decision-making and for future improvement of the staging system. The present research retrospectively assessed cases of GGN resection performed in Carmel Medical Center facility and compared clinical to pathological staging of the lesions. The present study will discuss the clinical meaning of this discrepancy and its effect on physicians’ decision-making and treatment approach.

## 2. Materials and Methods

### 2.1. Study Design

Data of 65 patients with 68 GGN type lesions, who were operated upon in Carmel Medical Center between 1 September 2015 and 31 December 2022, were retrospectively collected. All cases were discussed in a multidisciplinary meeting prior to the decision to proceed with surgery. Clinical staging was established based on the latest CT scan performed before surgical intervention. Surgical resection was thoracoscopic non-anatomical segmental resection in all cases. Prior to surgery, the lesions were CT–guided needle marked with a spiral hook wire (SOMATEX^®^ Lung Marker System, Berlin, Germany). Preoperative radiological clinical staging and postoperative pathological staging were compared. The local ethical committee approved the study. The study was conducted in accordance with the Declaration of Helsinki and approved by the Institutional Review Board of Carmel Medical Center (protocol code CMC-0027-23 on 1 January 2023). Patient consent was waived due to the retrospective nature of the study.

### 2.2. Data Analysis

Descriptive analyzes were reported by absolute values and proportions of the entire sample.

## 3. Results

### 3.1. Demographics

Demographic statistics of the study cohort are summarized in Table 1. The majority of patients were female and the median age for both male and female was 70 years.

### 3.2. Clinical and Pathological Staging

**Figure 1:**
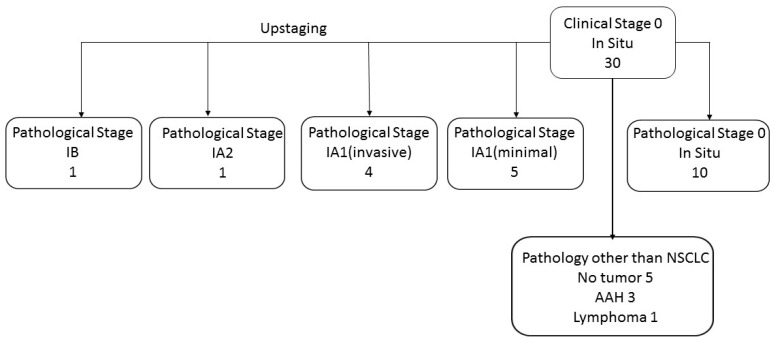
GGN clinical stage 0 distribution after final pathology report. (Abbreviations: AAH, atypical adenomatous hyperplasia).

In thirty cases, the radiological lesion was less than 3 cm in size and with no solid component. These lesions were defined as clinical in situ disease (Tis) and staged as stage 0 (Figure 1).

In twenty-one of the clinical in situ disease cases, the final pathology was non-small cell lung cancer. In five cases, no malignancy was found. In three cases, a premalignant lesion of atypical adenomatous hyperplasia was diagnosed and, in one case, a lymphoma was found.

Out of the twenty-one lung cancer tumors, eleven were upstaged (five tumors IA1 minimal, four tumors IA1 invasive, one tumor IA2, and one tumor IB) and ten tumors reserved their clinical stage on pathological staging.

For the twenty-one tumors that were clinically staged as in situ disease, stage 0, the mean and median radiological tumor size were 15.42 mm and 14.1 mm, respectively. In final pathology, the mean and median tumor size were 10.33 mm and 10 mm, respectively. This group of tumors were pure GGN; hence, the consolidation to tumor ratio was zero for all lesions.

In twenty-five cases, the radiological lesion was less than 3 cm in size with a solid component smaller than 5 mm. In those lesions, the T component was defined as clinical T1a minimal and the clinical stage was IA1 (minimal).

In twenty-two of the clinical IA1 (minimal) cases, the final pathology was non-small cell lung cancer. In two cases, no malignancy was found and, in one case, a colon carcinoma metastasis was found.

**Figure 2:**
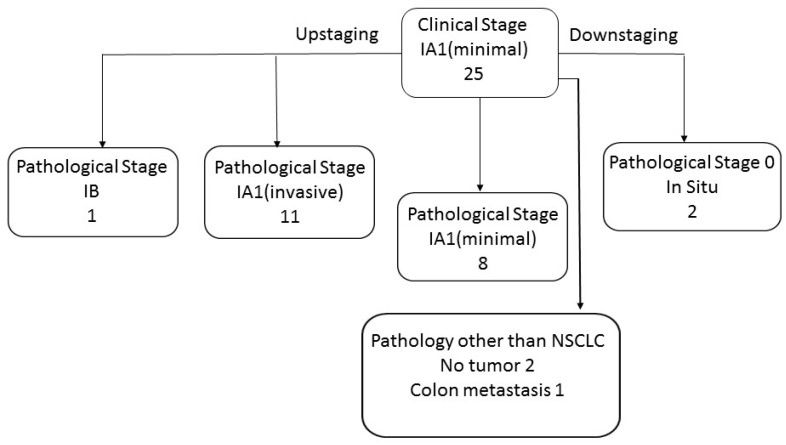
GGN clinical stage IA1 (minimal) distribution after final pathology report.

Out of the twenty-two lung cancer clinical IA1 (minimal) tumors, twelve were upstaged (eleven tumors IA1 invasive and one tumor IB) and two tumors were downstaged to stage 0 (in situ). Ten tumors reserved their clinical stage on pathological staging (Figure 2).

For the twenty-two tumors that were clinically staged as clinical IA1 (minimal), the mean and median radiological tumor size were 15.72 mm and 15.85 mm, respectively, and the mean and median solid component size were 3.35 mm and 3.45 mm, respectively. In final pathology, the average and median tumor size were 11.64 mm and 12 mm, respectively, and the average and median solid part size were 5.35 mm and 6 mm, respectively.

As for the consolidation to tumor ratio, in most lesions, sixteen, the ratio was less than 25%. In seven lesions, it was 25–50% and, in two, it was over 50%.

In nine cases, the radiological lesion was with a solid component of 5–10 mm. In those lesions, the T component was defined as clinical T1a invasive and the clinical stage was IA1 (invasive). One lesion was larger than 3 cm, but with no solid component, it was defined as lepidic predominant adenocarcinoma and staged as IA1.

In eight of the clinical IA1/IA1 (invasive) cases, the final pathology was non-small cell lung cancer. In one case, no malignancy was found and, in another case, a colon carcinoma metastasis was found.

**Figure 3:**
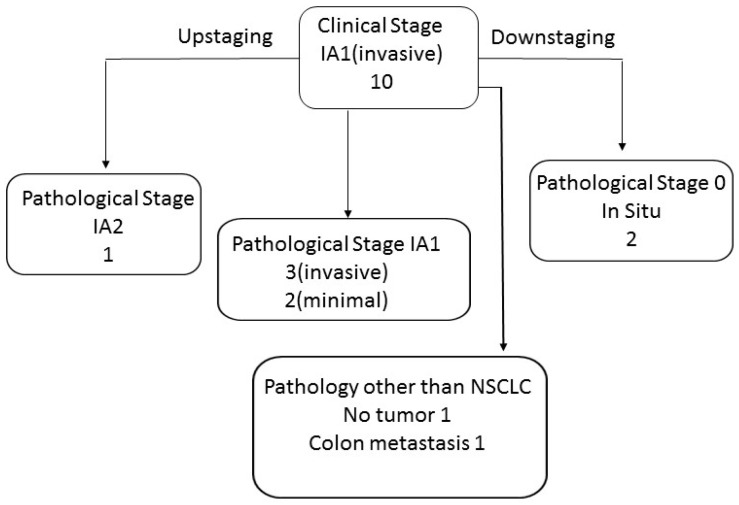
GGN clinical stage IA1 (invasive) distribution after final pathology.

Out of the eight lung cancer clinical IA1/IA1(invasive) tumors, one was upstaged (IA2) and two tumors were downstaged to stage 0 (in situ). Five tumors reserved their overall clinical stage IA1 on pathological staging (three remained IA1 (invasive) and two were defined as IA1 (minimal)) (Figure 3).

For the seven tumors that were defined as clinical stage IA1 (invasive), the mean and median radiological tumor size were 17.9 mm and 15 mm, respectively, and the mean and median solid component size were 7.85 mm and 8.3 mm, respectively. In final pathology reports, the mean and median tumor size were 11.35 mm and 12 mm, respectively, and the mean and median solid part size were 5.38 mm and 7 mm, respectively. For the one tumor that was defined as LPA, IA1 on clinical staging was measured on pathology as 25 mm overall size with an invasive part of 2.5 mm pathological stage IA1 (minimal).

As for the consolidation to tumor ratio, in most lesions, five, the ratio was more than 50%. In four lesions, it was 25–50% and, in one, it was zero.

Three lesions were defined as clinical stage IA2 due to a solid component 10–20 mm in size. On final pathology, two of those lesions were non-malignant and one was diagnosed as a carcinoid tumor.

Mediastinal lymph node sampling was performed in thirty-eight (56%) cases. No lymph node involvement was found.

Overall comparison of clinical to pathological staging is summarized on Table 2.

Over a third of the lesions, twenty-four (35.3%), were upstaged while only four (5.9%) lesions were downstaged. Another third of the lesions, twenty-three (33.8%), kept their stage. In three (4.4%) cases, premalignant lesion atypical adenomatous hyperplasia (AAH) was diagnosed. Ten (14.7%) cases were diagnosed as non-malignant on final pathology.

As for surgical data, no intraoperative complications were recorded. The postoperative course was uneventful for most cases. There were four cases of a prolonged air leak (over 5 postoperative days). The mean and median postoperative hospitalization period for all cases were 2.57 and 2 days, respectively.

## 4. Discussion

The approach to follow-up and treatment of GGN was reviewed in many studies and it still presents a clinical challenge [5,6,9,10,12]. The fact that these lesions tend to develop slowly over a long period (years) mandates a long period of surveillance. The 2017 Fleischner Society recommendations for a single pure GGN over 6 mm in size consistent on a CT scan at 6–12 months are to confirm persistence and then continue CT follow-up every 2 years until 5 years. For a single part solid GGN over 6 mm in size, a CT scan at 3–6 months is recommended to confirm persistence and then continued CT follow-up annually for 5 years if the nodule is unchanged; hence, the solid component remains under 6 mm [6]. If the GGN is smaller than 6 mm, usually no routine follow-up is necessary with the exception of a suspicious nodule (for example, morphology of lobulated margins or cystic components). These recommendations are based on studies such as the work of Kakinuma R. et al., which showed that the risk for a small (<5 mm) pure GGN to develop into invasive or minimally invasive adenocarcinoma is 1% over a mean follow-up period of 3.5 years [24]. During nodule surveillance, attention is set for changes that may correspond with malignant development: overall nodule enlargement, growing of the nodules’ solid component or thickening of the nodule haziness. Typically, a part solid GGN has a higher risk of malignancy than a pure GGN [5,6,9,10,11,12]. The power of a multidisciplinary team in evaluating such subtle changes is highly important. All patients in this study cohort were assessed by a multidisciplinary team that recommended, based on lesion morphology and changes during the follow-up period, surgical removal of the GGN.

Demographical data show that the study cohort had more female patients (60%); however, this had no statistical significance. The median age was 70 years for both gender groups, male and female.

Recent published work of the Task Force of the European Association of Cardio-Thoracic Surgery and the European Society of Thoracic Surgeons for the surgical management of GGN summarize therapeutic recommendations for these lesions [5]. These recommendations support non-anatomical segmentectomy (wedge resection) or anatomical segmentectomy depending on lesion location in the lung (peripheral versus central). Surgery should be performed via a minimally invasive approach. As for a lymph node, the recommendation for now is of systematic lymph node dissection that should be performed as part of staging, although it is not known to improve survival for pure GGNs and data for this specific issue are limited.

In all of the current study cases, the surgical intervention was thoracoscopic non-anatomical segmental resection after CT–guided needle marking with a spiral hook wire. There were no intraoperative complications. A postoperative prolonged air leak (over 5 days) was recorded in four (6.2%) patients and it was the only postoperative complication documented. It seemed that this postoperative complication has more to do with the specific lung characteristics of those four patients than with the procedure itself. The postoperative hospitalization period was short with a median of 2 days for all patients.

The dilemmas in the approach to GGN are reflected in the abundant number of guidelines by different societies as to how to follow-up and treat these lesions [5,6,11,25,26,27]. Most guidelines share some commonly agreeable concepts:

- Small pure GGN do not routinely need follow-up;
- Follow-up of a GGN should be over a considerably long period (years);
- The initial finding of a GGN should be determined by consecutive imaging in a suitable time frame;
- Subsolid GGNs have a much higher tendency to represent an invasive tumor;
- Lesions with a higher CTR correlate with a greater risk of invasiveness and nodal metastases;
- Lesion enlargement, consolidation or the development of a solid component are all incentives for further intervention during lesion surveillance;
- GGN biopsy is a conflicting predictor with a high percentage of false-negative results.

Still, other issues are more debatable, for example, what is the place of Positron Emission Tomography (PET) in GGN evaluation? Is there a GGN overall size or solid component size for subsolid GGNs from which surgical resection is highly recommended? When should a biopsy be taken from these lesions? What is the proper timing for intervention during GGN follow-up? For how long should a GGN be followed?

Tumor size, either overall or solid component size for a subsolid GGN, has long been a debatable issue as a cutoff indicator for intervention. In their review, Datterbeck FC and Homer RJ showed that the risk for a pure GGN to be adenocarcinoma in situ can reach 25% for lesions smaller than 10 mm and 40% for lesions larger than 10 mm. A higher percentage was noted for subsolid lesions. They concluded that a pure GGN larger than 10 mm or a subsolid GGN usually require intervention [28]. The American college of chest physicians practice guidelines state that pure GGNs larger than 10 mm that persist on follow-up should be biopsied or surgically resected. Subsolid GGNs larger than 15 mm should proceed directly to further evaluation with PET, nonsurgical biopsy and/or surgical resection [25].

In this study, pure GGNs’ clinical radiological overall size had a mean of 15.4 mm (median 14.1 mm) and subsolid GGN means were 15.7 mm (minimal, solid component < 5 mm) to 17.9 mm (invasive, solid component > 5 mm, median 15.8 mm to 15 mm). It seems from this analysis that 15 mm as an overall lesion size can be used as a guideline for more invasive assessment or intervention.

Considering the clinical preoperative stage, it appears that almost half of the GGNs, thirty (44.1%), were defined as adenocarcinoma in situ, AIS, stage 0. At this stage, the lesion was characterized as pure GGN, with no solid component. A higher clinical stage of IA1 was given to thirty-five (51.5%) of the GGNs. These lesions included a certain solid component and were part solid GGNs. Twenty-five of these lesions had a solid component smaller than 5 mm and were defined as minimal invasive adenocarcinoma, MIA, while ten nodules had a solid component larger than 5 mm (and <10 mm) and were defined as invasive adenocarcinoma. The remaining three nodules (4.4%) had a solid component larger than 10 mm (and <20 mm) and were, therefore, defined as clinical stage IA2.

Comparing the clinical to pathological stages reveals that over a third of the lesions, twenty-four (35.3%), were upstaged while only four (5.9%) lesions were downstaged. Another third of the lesions, twenty-three (33.8%), kept their stage. In three (4.4%) cases, premalignant lesion atypical adenomatous hyperplasia (AAH) was the conclusion of the final pathology. These findings show an overall low agreement between the clinical to pathological stages of GGNs.

Another significant group of GGNs in this study is the ten (14.7%) cases that were diagnosed as non-malignant on final pathology. Those patients retrospectively did not mandate resection. This group demonstrate the sometimes obscure nature of GGNs and the difficulty in decision-making as to surgical intervention versus follow-up.

When assessing GGNs, which present clear indicators of tumor invasiveness and grow during surveillance, the decision upon intervention is relatively simple, but for unchanged persistent pure GGNs, the choice between intervention and to continue follow-up is more difficult. These cases encourage physicians to use more vague indicators and incentives for intervention, such as tumor size, patient lung characteristic, nodule characteristic, patient medical status, etc. If the uncertainty remains very high, the slow-growing nature of GGNs allows postponement of the decision while continuing follow-up.

This work attempts to address GGNs from a less common perspective, i.e., clinical versus pathological staging. Data analysis of this study cohort implies that the decision upon surgical intervention should be taken very thoughtfully. The relatively high percentage of upstaging tumors and overall safe and short surgical procedure advocate for surgical resection even in the presence of a significant number of non-malignant lesions that retrospectively do not mandate intervention at all. This somewhat conflicting dilemma requires patient sharing prior to consent, with a genuine effort to explain this complex situation.

## 5. Conclusions

Decision-making during GGN surveillance requires a significant clinical judgment, hence, the power of a multidisciplinary team that evaluate and follow this type of lesion. This clinical study, although it has some inherent disadvantages (retrospective and a relatively small number of cases), adds to the growing volume of data on these lesions that may be encountered more often in the following years.

## Figures and Tables

**Table 1:** Demographic characteristics of the study population (*n* = 65).

Gender	Frequency (Percentage), *n* (%)	Median Age (Years)	Mean Age (Years) ±SD
Female	39 (60%)	70	69.4 ± 6.61
Male	26 (40%)	70	68.5 ± 9.49

**Table 2:** Comparison between the clinical and pathological stages of GGNs (*n* = 68).

Clinical Stage	In Situ *n* = 30	IA1 (Minimal) *n* = 25	IA1 (Invasive) *n* = 10	IA2 *n* = 3	Total Nodules *n* = 68 (%)
Pathological Stage *					
*No Tumor*	*n = 5*	*n = 2*	*n = 1*	*n = 2*	*10 (14.7%)*
*No NSCLC*	*n = 1*	*n = 1*	*n = 1*	*n = 1*	*4 (5.9%)*
*AAH*	*n = 3*	*n = 0*	*n = 0*		*3 (4.4%)*
*Same Stage*	*n = 10*	*n = 8*	*n = 5*		*23 (33.8%)*
*Downstaging*	*n = 0*	*n = 2*	*n = 2*		*4 (5.9%)*
*Upstaging*	*n = 11*	*n = 12*	*n = 1*		*24 (35.3%)*

* Pathological staging data are presented in italics. Abbreviations: GGN, ground glass nodule; NSCLC, non-small cell lung cancer.

## Data Availability

The original contributions presented in this study are included in the article. Further inquiries can be directed to the corresponding authors.
